# Bottom-Up Self-Assembled Supramolecular Structures Built by STM at the Solid/Liquid Interface

**DOI:** 10.3390/ma12030382

**Published:** 2019-01-25

**Authors:** Quirina Ferreira, Catarina L. Delfino, Jorge Morgado, Luís Alcácer

**Affiliations:** 1 Instituto de Telecomunicações, Instituto Superior Técnico, Av. Rovisco Pais, 1049-001 Lisboa, Portugal; catldelfino@gmail.com (C.L.D.); jmorgado@lx.it.pt (J.M.); alcacer@lx.it.pt (L.A.); 2 Department of Bioengineering, Instituto Superior Técnico, University of Lisbon, Av.Rovisco Pais, 1049-001 Lisbon, Portugal

**Keywords:** scanning tunneling microscopy, unimolecular electronics, molecular device, monolayer, coordination chemistry, interfaces, nanotechnology

## Abstract

One of the lines of research on organic devices is focused on their miniaturization to obtain denser and faster electronic circuits. The challenge is to build devices adding atom by atom or molecule by molecule until the desired structures are achieved. To do this job, techniques able to see and manipulate matter at this scale are needed. Scanning tunneling microscopy (STM) has been the selected technique by scientists to develop smart and functional unimolecular devices. This review article compiles the latest developments in this field giving examples of supramolecular systems monitored and fabricated at the molecular scale by bottom-up approaches using STM at the solid/liquid interface.

## 1. Introduction

The development of functional and smart organic materials for optoelectronics has been a challenge for researchers in the field of nanoengineering. The interest lies in the possibility of designing and developing materials with specific functionalities and integrating them in electronic devices, such as sensors [1], light-emitting diodes [2], photovoltaic cells [3,4] and transistors [5,6]. Research has been focused on low-cost and versatile methods of material production combined with the necessity of making smaller and denser electronic devices. Molecular electronics is a promising solution to overcome this challenge using individual molecules or molecular assemblies as the active components of electronic circuits [7,8]. Over the last decade, expectations for this field were driven by (i) the possibility of these molecules becoming alternatives to silicon-based technologies, which is the at the core of the integrated electronic circuits; (ii) several synthesis and manufacturing processes that have already been adapted and several techniques made available that allow the preparation of high quality molecular films and the characterization of these devices; (iii) the ability to design and model structures at a molecular level; and (iv) the application and integration of molecular devices in real-life applications.

A molecular device is an assembly of molecular elements designed to behave in a certain manner, operating in a specific capacity and developed for a precise function. Each element contributes with a single function cooperating for the performance of the complex molecular device. The individual components can operate as a result of an external stimulus and can be easily replaced by other elements to accomplish a desired function. Each element can be individually stimulated as a result of an external source. This idea was introduced for the first time by Drexler [9]. He proposed a nanodevice construction using a stepwise method adding atom by atom until a complex supramolecular structure was achieved, to which he called “assembler”. This idea has been implemented and revolutionized the manufacture of electronic devices opening the door to new complex structures that have never been developed because the traditional top-down methods do not allow it. Following the experiments of Pederson [10], Cram [11] and Lehn [12], who shared the Nobel Prize in Chemistry in 1987, on supramolecular chemistry, new molecular devices have been developed by a bottom-up approach, connecting self-assembled molecules to obtain larger structures. Recently, the winners of Nobel Prizes in Chemistry 2016, Sauvage [13], Stoddart [14] and Feringa [15] showed how it is possible to build and manipulate nanomachines. Thereby consolidating the idea that the molecule by molecule approach allows the fabrication of complex electronic devices.

The development of materials at the molecular level requires specific fabrication techniques that allow visualization and manipulation of matter at this scale. This is the point that this review article aims to address, showing how molecular devices can be fabricated using a bottom-up approach based on atom-by-atom or molecule-by-molecule assembly until a functional and complex structure is achieved. Scanning Tunneling Microscopy (STM) is the elected technique to play with matter at the atomic level. Namely, STM has been used to monitor in real-time formation of highly organized self-assembled monolayers where the molecules arrange themselves into packed 2D crystals fully covering the surface of the substrate onto which they are deposited. Several high-resolution STM images of the formation of molecular systems by self-assembly have been reported revealing the parameters involved in the monolayer formation: whether they are physical parameters (e.g., concentration of solutes, wettability, contact angles, surface tension, temperature) or chemical parameters (e.g., molecular structure, orbital configuration). All these monolayer properties are important for the design of organic devices.

**Figure 1:**
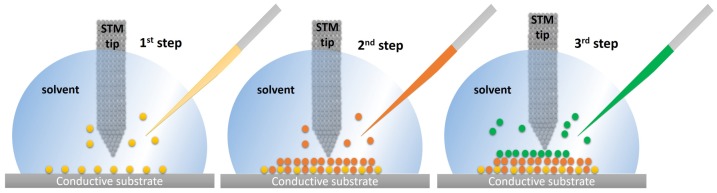
Schematic representation of a stepwise building of supramolecular structures by STM at the solid/liquid interface. Each step corresponds to one molecule or molecular system which can be added individually and monitored by high-resolution imaging. The experiments occur inside a drop of a high boiling point solvent which is added on the top of a conducting substrate and allows the phased addition of molecules.

Recently, STM has been used to build molecular systems with multicomponents, e.g., self-assembled monolayers with more than one molecular element or vertical supramolecular structures synthesized in situ [7,8,15,16]. STM experiments can be carried out at the solid/liquid interface, meaning that take place at the interface between a high boiling point solvent when they occur at the interface with a high boiling point solvent which is added to a conducting and atomically flat substrate. Ideally, this drop should be preserved during long periods of time (several hours) to allow enough time to image and monitor a monolayer in real time. The drop works as a nanolab where the molecules can be added to form one or more monolayers, as schematized in Figure 1. This nanolab allows the in situ synthesis of complex supramolecular structures, where each step can be monitored by high-resolution imaging [7,8]. Each system requires specific conditions: nature of the substrate, solvent, temperature, concentration, and characteristics of the tip. The solvent has an important role, since it can participate in the formation of the monolayer coadsorbing with the molecules that are intended to be deposited [17].

## 2. Molecular 2D Patterned Arrays

Conducting organic self-assembled and self-organized monolayers are the starting point of an organic device fabrication. They are spontaneously formed on a substrate, usually on a conducting substrate, and they can be composed of one or more than one type of molecules, depending on the aimed application. Due to the high reproducibility of this technique, it is easy to use self-assembled materials in the development of large-scale devices. The molecular ordering is strongly dependent on the intra- and intermolecular interactions, temperature, concentration, solvent and on the chemical/physical interactions with the substrate ([18] and the references in there). The self-assembly and self-organization properties of molecular systems are influenced by the substituents which in turn can originate steric [19,20] and electronic effects [21,22] that affects the molecular array. The stability of these systems is, in most cases, ensured by non-covalent bonds established by the subunits. Hydrogen, halogen, and coordination bonds are the support of most solid-state structures and they are the forefront in the design of these systems. With the introduction of graphene as a substrate of self-assembled monolayers, π–π interactions have been receiving particular attention from scientists, since they are responsible for many molecular arrangements [23]. STM is the perfect tool to study the influence of all these factors while monitoring the monolayer formation in real time through high-resolution imaging. The fabrication of several monolayers has been reported using STM to image the molecular organization and in some cases to manipulate their formation. Experiments at the solid/liquid interface have contributed to the development of organic devices at room temperature, under atmospheric pressure and in some cases in aqueous environments. This represents an important progress on the development of materials at a sub-molecular scale taking into account that most of the devices operate in these conditions.

### 2.1. Polymorphism

**Figure 2:**
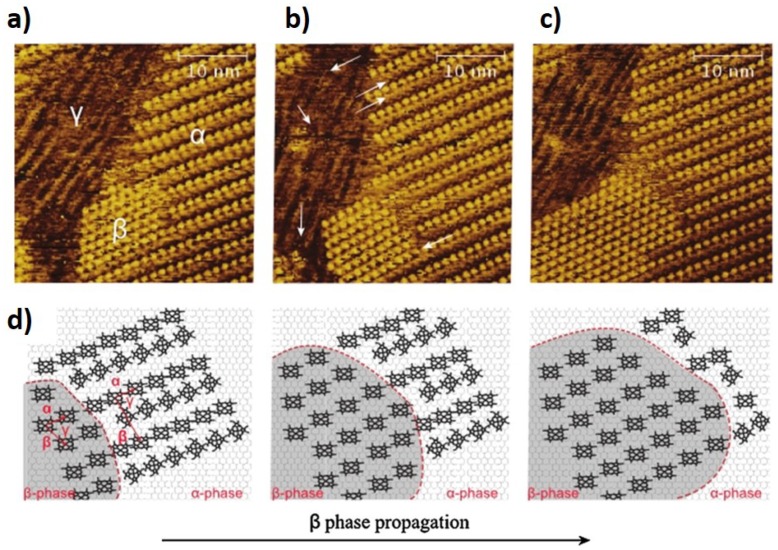
STM images (**a**–**c**) of ZnOEP adsorption on highly oriented pyrolytic graphite (HOPG) were recorded sequentially with 2-min intervals, starting 2 h after the deposition of porphyrin in the tetradecane droplet. These STM images show a polymorphic monolayer with three different co-existing phases, in which the white arrows point to molecules with increased contribution to the signal and a propagation of a β-phase, which is modelled in (**d**). (Reprinted with permission from reference [17]. Copyright 2013 Elsevier B.V)

The formation of a monolayer can have intermediate metastable phases with a molecular packing configuration different from that of equilibrium [24,25,26,27,28]. In particular, the monolayers formed at the solid/liquid interface are influenced by the solvent, which in turn can induce polymorphism in the molecular organization. In some cases, the solvent molecules are co-adsorbed with the molecules that are being deposited. In particular, competition between the molecules being deposited and the solvent occurs as the monolayer forms at the solid/liquid interface. Some of these molecules are adsorbed together with the solvent molecules. Competition with the solvent can be seen from the moment that the molecules are being adsorbed on top of the substrate. Ferreira et al. used STM to visualize the competition effect between zinc porphyrins and tetradecane for more than 2 h [17]. Figure 2 shows consecutive STM images acquired 2 h after deposition of the molecules, evidencing the coexistence of three metastable phases α, β and γ which correspond to different molecular packing associated with different conformations of porphyrins. The authors attribute the coexistence of these three phases to a competition between the porphyrins and the solvent.

In solid/liquid interface experiments, the equilibrium of a packed monolayer depends on the adsorbed/desorbed molecules and the molecules that remain in the liquid environment [29]. To achieve the stability of the molecular organization, molecules that are being adsorbed compete with the molecules that remains in solution. Consequently, the molecular packing can change from a dense array to a porous packing, as well as from a fully packed monolayer to a disorganized array [30,31].

Polimorphism is important in the design of organic devices, since it affects the carrier transport [32]. The analysis of molecular conformation changes in real time is essential to identify metastable phases. Scanning probe microscopies, mainly the STM and the atomic force microscopy (AFM) [33,34,35,36,37] can image the evolution of monolayers and allow to understand the factors that influence the stability of self-assembled systems.

### 2.2. In Situ Synthesis

Two-dimensional self-organized monolayers can work as anchor points to obtain vertical structures, bonding molecules one by one. Otsuki et al. [16] described the fabrication of labile axial ligands (i.e., pyridine) on a template monolayer of zinc porphyrins, deposited beforehand on highly oriented pyrolytic graphite (HOPG). They prepared ordered arrays of porphyrins (H${}_{2}$(C${}_{18}$OPP)) and metalloporphyrins (Zn(C${}_{18}$OPP)) at a HOPG/1-phenyloctane interface, on top of which an azo-functionalized molecule (4-(phenylazo)pyridine)) was assembled via axial coordination to the metallic center, both in a *cis-* and in a *trans*-conformation. STM data of the porphyrin films did not provide differentiation until addition of azo molecule.

**Figure 3:**
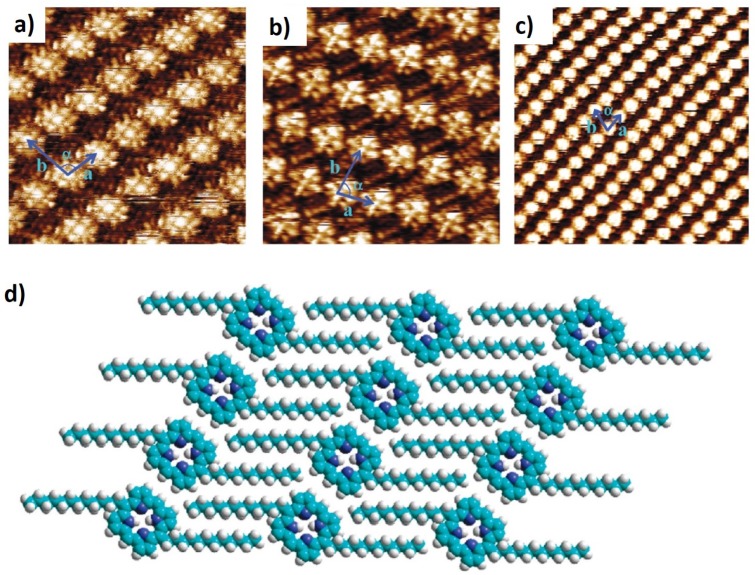
STM images of different self-assembled monolayers: (**a**) monolayer of non-coordinated 5,10,15,20-*meso*-tetradodecylporphyrin (TDP) (8.1×8.1 nm${}^{2}$); (**b**) monolayer of the same porphyrin coordinated with a zinc metal-ion (ZnTDP) (9.3×9.3 nm${}^{2}$); (**c**) a monolayer of 3-nitropyridine molecules that were self-assembled and coordinated to the layer underneath, a ZnTDP monolayer (20.8×20.8 nm${}^{2}$); (**d**) packing arrangement model for TDP assembled on HOPG. (Reprinted with permission from reference [38]. Copyright 2016 American Chemical Society).

A few years later, Feringa and co-workers [38] reported on the axial construction of suprastructures with an in situ self-assembly strategy, relying on metalloporphyrin arrays as building units. More specifically, a Zn-tetrakis(meso-dodecyl)porphyrin (ZnTDP) self-assembled monolayer was deposited at the HOPG/n-tetradecane interface and covalently bonded to a meta-substituted pyridine, namely 3-nitropyridine. ZnTDP and the pyridine were also placed together in solution to ensure that axial coordination between the molecules would only occur once the porphyrin monolayer would assemble at the interface. Prior to STM imaging the compounds were dissolved in a specific solvent (n-tetradecane) and then a droplet of solution was applied on freshly cleaved HOPG and imaged with STM at room temperature. Upon physisorption of the molecules at the interface, high-resolution STM images of well-ordered patterned arrays were obtained. Figure 3c shows the monolayer formed by the porphyrin covalently bound to the pyridine. After the self-assembly of the monolayer each porphyrin lies flat at the interface by π–$\pi^{*}$ stacking over the HOPG substrate. The authors observed a higher ratio of coordinated porphyrins compared with unreacted porphyrin. That was explained by the preference of pyridines with physiosorbed porphyrins, rather than with the porphyrins in solution.

This work paved the way to in situ synthesis of supramolecular systems and unimolecular devices [7,8] developed by a layer-by-layer approach.

### 2.3. Nanoporous Networks

Two-dimensional nanoporous networks consist of self-assembled monolayers with a porous pattern which can host small molecules. The inclusion of guest molecules is dependent on the size and/or shape of the pore (physical inclusion) or on the molecular recognition (chemical inclusion). The porous array can be modified for specific purposes using rational molecular design. Two methodologies are available for the fabrication of nanoporous surfaces depending on the type of building blocks: intrinsic and extrinsic [39,40]. Nanoporous monolayers developed with intrinsic building blocks are based on macrocyclic molecular structures that are pre-designed with a hollow center and synthesized beforehand and with specific physical and chemical characteristics. Upon deposition, they retain the designed properties for each intrinsic nanopore, e.g., pore size. Circular molecular structures that can be used as intrinsic building blocks can occur in nature, e.g., proteins and protein complexes, or be specifically synthesized with a hollow center, as crown ethers, porphyrins, and phthalocyanines.

Nanoporous monolayers developed with extrinsic building blocks depend on the self-assembly of acyclic molecules that together form extrinsic pores. Among the most used extrinsic building blocks we find trimesic acid (TMA) [41,42], dehydrobenzo[12]annulene (DBA) [43,44,45,46,47] and dicycanooligophenylenes [48,49]. They form hexagonal nanopores in which the pore size can be modulated by changing the chemical composition of each building block [39,40,41,45]. These are the most attractive systems, since it is possible to modify the pore properties using specific building blocks to obtain a desired purpose. The fabrication of flat well-defined nanoporous surfaces is not trivial and the deposition conditions, tailored to a goal, must be optimized. To ensure that the deposited molecules retain a specific packing arrangement avoiding pore hinderance or self-assembly of flat-lying monolayers it is important to know the interactions between molecules and surface. Table 1 summarizes the main interactions of these extrinsic building blocks that were analyzed in nanoporous self-assembled networks [39].

The solvent can play an important role in the formation of nanoporous networks. Lackinger et al. [41,42] demonstrated how the solvent can induce different types of nanopores. The authors reported the formation of a self-assembled nanoporous monolayer using TMA as an extrinsic molecular building block that forms two polymorphs influenced by the presence of alkanoic acids that were used as solvent. They analyzed the influence of alkyl chain length of the alkanoic acids, from butyric to nonanoic, on the molecular arrangement of TMA molecules in the presence of the different solvents. They observed that the solubility decreases as a function of the increase in the alkyl chains length and a “flower” pattern was observed for shorter acids (butyric to hexanoic acid) while a “chickenwire” was obtained for octanoic and nonanoic acids. The obtained pores have the same dimensions for both structures (about 1 nm) but the center-to-center distance between pores is 1.7 nm for the “chickenwire” and 2.5 nm for the flower arrangement.

**Figure 4:**
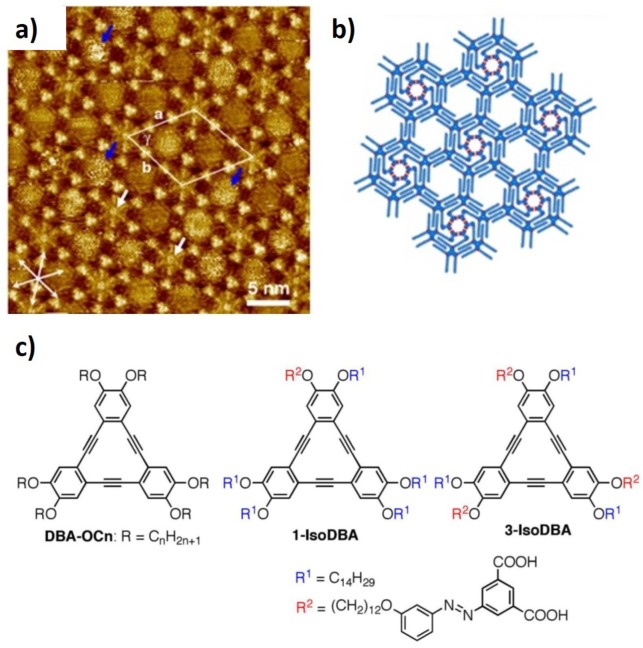
(**a**) STM image of a self-assembled monolayer of DBA functionalized with isophthalic acid linked to the alkyl chains by azobenzene groups. The image shows a honeycomb array characterized by two types of pores: smaller ones indicated by white arrows surrounded by the isophthalic units and larger ones indicated by blue arrows involved by the interdigitated alkyl chains. (**b**) molecular model of the DBA monolayer observed in (a). (**c**) chemical structure of DBA-OCn molecules and the DBA molecules functionalized with isophthalic units (1-IsoDBA and 3-IsoDBA) (Reproduced with permission from reference [43] Copyright from 2016 American Chemical Society).

A second class of extrinsic building blocks are alkyl-/alkoxy-hexadehydrotribenzo[12]annulene (DBA) molecules (see chemical structure in Figure 4c that allow the formation of reproducible nanopores [45,46,47,51]. They have alkyl chains as substitutes which allows the interdigitation of chains between neighboring DBA molecules. Interdigitation is dependent on chain length, type of solvent, concentration of DBA molecules and temperature [46,51]. By controlling these parameters, the authors were able to design specific features for the resulting nanopore network, such as, the nanopore diameter and the unit cell of the array. The stabilization of the honeycomb array can be ensured by introducing solvent molecules which have a high affinity with the substrate and that coadsorb with the DBA molecules (“template-induced” method) or controlling the concentration of molecules in solution (“concentration-in-control method”). The authors reported that a diluted solution of DBA molecules with shorter alkyl chains in a good solvent, at high temperature tend to form a more densely packed structure. Additionally, at high concentration of DBA molecules in 1,2,4-trichlorobenzene (TCB), porous networks were only achieved for $DBA-OC_{10}$ and $DBA-OC_{12}$ (chemical structure in Figure 4, contrariwise $DBA-OC_{14}$ and $DBA-OC_{16}$ yielded non-porous monolayers. DBA-OCns nanoporous networks can exhibit pore diameters from 1.6 nm to 4.7 nm, depending on the length of the alkyl chains.

A functionalization of the alkyl chains of DBA molecules allows the modulation of their physical and chemical characteristics of the nanopores, i.e., pore size, shape, and electrostatic properties. Tahara et al. [44] reported on an equally functionalized network built with the ability to tailor nanoporous size reversibly by exposing the array to a specific radiation wavelength. The authors developed a DBA self-assembled nanoporous array, in which the DBA molecules were functionalized with a photoresponsive azobenzene group, to yield azobenzene-functionalized DBA networks. Upon the formation of the array, the azo group can be stimulated by light to induce a conformational change in the DBA network, uniformly establishing a new nanoporous network. Nanoporous arrays can also be composed of contrasting motifs within the same array, since sections of the array can be functionalized differently, i.e., being periodically functionalized. The authors achieved this by using non-isotopological DBA molecules [43]. Non-isotopological DBA-based arrays, with a honeycomb structure, can be assembled with functionalized and unfunctionalized nanowells, achieving a network array with distinct pore sizes. This was obtained after annealing a monolayer of isophthalic acid units connected by an azonbenzene linker to the alkyl chains on top of HOPG. Figure 4 shows a STM image of the obtained monolayer at HOPG/1,2,4-trichlorobenzene interface which presents a honeycomb structure. It is possible to observe white triangular features that represents the core of the DBA molecule and darker regions representing the interdigitated chains extending from the DBA cores. Two types of nanopore are formed and indicated by the blue (larger pores) and white (smaller pores) arrows. They are also represented by the molecular model in Figure 4b.

Dicycanooligophenylenes are another class of building block that form isotopological hexagonal packings that are governed by metal-ligand coordination bonds [48,49]. The reported works were not developed at ambient conditions and the authors used ultra-high vacuum (UHV)to obtain the organized network. Despite that, these systems can be explored to be used at ambient conditions.

The design of nanoporous surfaces has been a challenge due to the several applications of these systems [39]. The inclusion of guest molecules is another important point to be taken into account, since in some cases, their chemical and physical properties are changed after inclusion in the nanoporous network.

### 2.4. Host-Guest Systems

Molecular networks can integrate guest single molecules, homoclusters, or heteroclusters. Host-guest systems can be used in their simpler form or be chemically modified, thereby allowing modulation and functionality control of the interior of each nanopore conferring more versatility to the host matrices [39]. The combination of rationally designed 2D nanoporous networks with host-guest chemistry is valuable for a diverse range of applications, namely tailored catalysis. For this application, shape and size of each nanoreactor are crucial for binding. Host-guest dedicated requirements are comprised of sensitivity, selectiveness, and stimuli-responsiveness upon binding. The nanopore size and shape also require the consideration of complementarity. Nevertheless, the challenge in the design and synthesis of host molecules relies, not only on the host-guest chemistry, but also on the stability and the characteristics of the host network itself. Lackinger [42] and Griess et al. [52] reported that a porous TMA host matrix of 1.3 nm average pore size is ideal to accommodate planar and spherical guest molecules of similar sizes (e.g., coronene, sunflower, $C_{60}$, and triangular nanographene (TNG)) via van der Walls interactions between substrate and host matrix.

Simple DBA host networks can also be modelled, accommodating different guests, in size by changing the length of the alkyl chains and the concentration of the guest. Balandina et al. [53], showed that the length of alkyl chain of the host, the solvent, and the concentration of guest molecules influences the equilibrium of the system. Considering coronene (COR), as a guest, the authors reported that one COR molecule could be fitted in a DBA-$OC_{6}$ nanowell network, while for networks of DBA-$OC_{10}$, COR is coadsorbed with solvent molecules. For longer alkyl chains (DBA-$OC_{14}$ and -$OC_{16}$) a phase transition of the host structure is induced by an increase of concentration of guest COR molecules, consequently affecting the equilibrium of the incorporation. Furthermore, a different number of incorporated guests can be seen when considering triangular nanographene compounds as triangular-like shaped guests for the same DBA-type network. Lei et al. [51,54] observed the incorporation of up to six TNG molecules into different hexagonal porous networks of DBA molecules composed of alkyl chains with chain length ranging from $OC_{10}$ to $OC_{20}$ [39].

**Figure 5:**
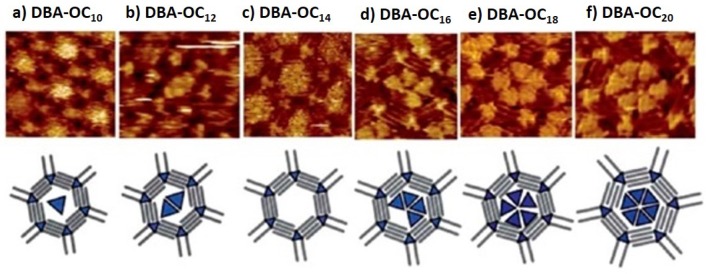
STM images of nanoporous monolayers developed using DBA building blocks with different chain lengths (**a**) DBA-$OC_{10}$, (**b**) DBA-$OC_{12}$, (**c**) DBA-$OC_{14}$, (**d**) DBA-$OC_{16}$, (**e**) DBA-$OC_{18}$, and (**f**) DBA-$OC_{20}$, and therefore consequently different pore sizes for a host-guest network of (DBA-OCns)-TNG. A schematic model of the host-guest systems is represented below of the corresponding STM image. Different nanowell sizes can incorporate different numbers of a guest molecule (TNG). (Reprinted with permission from reference [51]. Copyright 2010 The Royal Society of Chemistry).

Figure 5 represents the dependence of the number of triangular shaped guests on the nanopore size. Comparing Figure 5 from (a) to (f) we notice a linear increase of guest molecules adsorbed as a function of DBA alkyl chain length. Namely, for the DBA-$OC_{20}$ we observe a total of six TNG molecules neatly packed inside of DBA pore. The respective STM image shows bright triangular domains organized as a hexagonal motif that is commensurate with the DBA pore shape. An exception was observed for the DBA-$OC_{14}$ (Figure 5c) due to a phase transition that occurred only for this case, in which, the TNG molecules were too mobile to be visualized and are described by the authors as “fuzzy features” present in the STM image at the center of each nanowell. Comparing the incorporation of both guest molecules into the same type of DBA network, gives some insight into the effects of using different guests and their impact on the host-guest system properties. For example, the shape of the guest affected the shape complementarity with the hexagonal nanowells, i.e., being more commensurable, generally led to a higher number of TNG molecules being adsorbed when compared to COR. Also contributing to this effect was the increased affinity of TNG towards HOPG, which was contributed to an increase number of more guests being adsorbed per nanowell.

**Figure 6:**
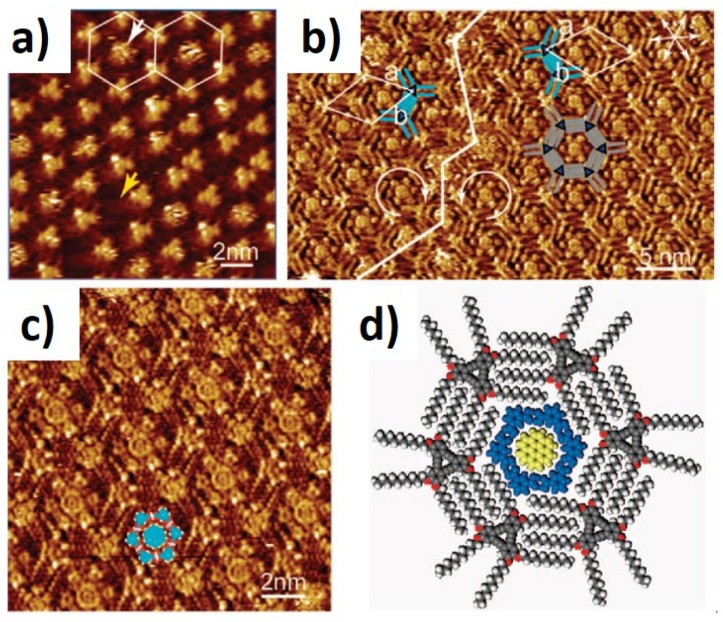
STM images of nanoporous monolayers developed using DBA-$OC_{10}$ building blocks at the HOPG/1-octanoic acid interface. The DBA host network can be visualized with (**a**) and without (**b**) a guest molecule (an heterocluster of ISA and COR); (**c**) high-resolution STM image of (b); (**d**) schematic representation of one host-guest system. (Reprinted with permission from reference [55]. Copyright 2008 The American Chemical Society).

More than one guest “molecule” can be adsorbed at the same time as an heteromolecular cluster. Lei et al. [55] used a complex formed by COR and isophthalic acid (ISA), in which COR is surrounded by six ISA molecules, as a guest heterocluster to be adsorbed in a DBA network. For this case, the increase of pore size does not correspond to a linear increase of incorporated guests. Observing the STM images of Figure 6 is possible to observe that the molecular organization of the incorporated cluster represented by the circular brighter domains surrounded by six smaller triangular motifs corresponding to the DBA molecules. The effect of the alkyl chains length was analyzed and only the monolayer composed of $DBA-OC_{10}$ was able to neatly incorporate the cluster. DBA networks with smaller chain lengths, originate smaller pore sizes, which do not allow the complex to go inside. For a bigger nanopore size the cluster has higher mobility affecting the acuity of STM images acquisition.

Host-guest chemistry via charge-transfer can also be implemented using porphyrins at the solid/liquid interface, as reported by Iriani et al. [56] using a zinc porphyrin (DBA-ZnP). The DBA-ZnP nanoporous network assembled at the HOPG/TCB interface can accommodate $C_{60}$ fullerene.

Host-guest chemistry may assist the catalytic chemical transformations that take place in a nanoreactor. It has the advantage of adjusting the physical and chemical properties of each nanoreactor space. The intercalation of organic molecules into molecular suprastructures is a promising approach in the field of nanoreactors fabrication. Nanoporous network arrays, if properly equipped, as nanoreactors can through chemical interactions capture guest species and reagents involved in the chemical transformation [39,50,57,58].

## 3. Molecular Machines

A molecular machine is an assembly of molecular elements that has mechanical movement when stimulated by external inputs. Comparing macroscopic with molecular-level machines, both are characterized by an energy source required to achieve a specific function, and by the manner through which their operation can be monitored. Nevertheless, motion laws are different and consequently the design and manufacturing methods are specific to each scale. Macroscopic machines are described by Newton’s equations that cannot be applied to the nanoscale world. With the development of powerful scanning probe microscopies, mainly, with the contribution of STM, the movement of single molecules can be controlled and characterized. All living organisms are composed of thousands of molecular machines, biomolecular machines, that ensure the correct operation of all functions. The way they work can inspire engineers to develop powerful synthetic molecular machines. The concept of molecular machines was fostered by the winners of the Nobel Prize in Chemistry of 2016 [15,59,60,61] who inspired the world of scientists that work in nanotechnology to design molecular machines that move in response to an external stimulus. Feringa [62] describes, a nanoworld composed of factories with molecular dimensions which that are self-repairing, operating with molecular precision and driven by light and chemical energy. Feringa explains that the major difficulty is not to achieve the motion of molecular machines, but to control their operation and maintain their stability to ensure directionality. The central part of any molecular machine is the motor, and scientists are focused in developing molecular motors able to operate at room temperature. The following sections of this paper introduce the best-known synthetic molecular machines, molecular switches, and motors, built and controlled by STM.

### 3.1. Molecular Switches

An electronic circuit is managed by switches that control current flow. A molecular switch is a molecule or a supramolecular system that can exist in two states, ON and OFF, accessible by an external stimulus. STM has been a commonly employed tool to characterize and to stimulate these structures. The tip is the central part of the experiment and can switch a molecular system between two states in a reversible way. Most of these studies are at solid/liquid interfaces and allow the characterization of individual electrical properties of both states, ON and OFF. They can be grouped in different categories depending on the stimuli received: electronic, photochromic, or mechanically interlocked [63]. The switching properties are dependent on the electrode’s composition, the molecular structure, and the environment where the experiments are done. The stimuli involved in these experiments are the electrons, which flow from the STM tip, and can consequently induce: desorption and/or dissociation of molecules from a surface, hopping effects, molecular rotation and in situ chemical reactions [64].

**Figure 7:**
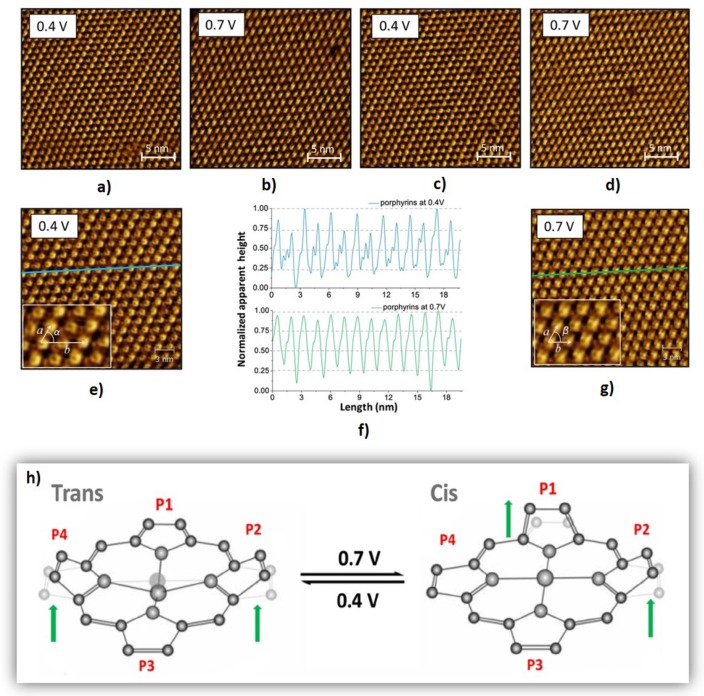
(**a**–**d**) Sequential STM images of ZnOEP monolayer at the tetradecane/HOPG interface revealing conformational changes upon application of stimuli. (**e**) the blue line and (**g**) the green line acquired from the STM images are represented (**f**) by profile lines in normalized apparent height maps indicating the differences between heights in accordance with different molecular conformations. (**h**) Reversible switch able mechanism of ZnOEP molecules representing the Trans and Cis conformations. The pyrrole groups indicated by P1, P2, P3, and P4 adopt different positions in relation on the plane of the macrocycle. The green arrows indicate the pyrrole groups which are turned up. (Reprinted with permission from reference [65]. Copyright 2018 The authors and Royal Microscopical Society).

Most of the experiments occur at low temperatures and under ultrahigh vacuum to avoid thermally induced effects. However, most of the times, these conditions do not represent the real environment of the molecular device, mainly in biological and organic electronic devices. Marbach et al., induced a conformation of porphyrins presented in a self-assembled monolayer close to room temperature (200 K). STM images of a self-assembled porphyrins monolayer on Cu(111) showed different molecular conformations of porphyrins as a function of the applied tip-voltage. Recently, Ferreira and co-workers [65] reported a switchable mechanism of a self-assembled monolayer of porphyrins on HOPG at room temperature induced by STM. Figure 7 shows the dynamics of the porphyrins presented in this monolayer through consecutive STM images acquired in the same region at constant current and at alternate tip potentials of 0.4 and 0.7 V. Figure 7e unveil two types of porphyrins well distinguished by the apparent height in the respective line profile of Figure 7f. The authors attributed these differences to different molecule conformations associated with different pyrrole group positions. Figure 7h indicates what happens to the pyrrole groups at each conformation. At 0.4 V, the molecules present a Trans conformation characterized with the P2 and P4 pyrroles turned up in relation to the plane of macrocycle, as is indicated by the green arrows. The pyrrole groups adopt a different behaviour when a bias voltage of 0.7 V is applied, and the porphyrins adopt a Cis conformation characterized by consecutive pyrrole groups turned up (see the P1 and P2 indicated by green arrows). The STM images acquired in the same region and at alternate potentials support this switchable and reversible mechanism.

The pyrrole groups of brighter lines are in Trans conformation and the darker lines correspond to a Cis conformation. When the tip-voltage increases to 0.7 V the Trans molecules switch to a Cis conformation and the monolayer becomes homogeneous as it is indicated by the line profile of Figure 7f. It was observed that the response of porphyrins to the tip-voltage is reversible and does not cause disorders in the self-assembled monolayer. This work shows the stability of self-assembled molecular switches at room temperature and supports the idea that they are great candidates to connect with other molecules in a complex electronic circuitry.

### 3.2. Molecular Motors

Movement is essential to life, may it be in the form of transport, translocation, contraction, or others, since ultimately biological systems rely directly or indirectly on movement. No wonder molecular motors, which have the ability of powering movement, are intrinsic to nature. Molecular motors are commonly seen in nature as, for example, in adenosine triphosphate synthase, myosin, kinesin, and dynein. These are motor proteins that use chemical energy to attain directional movement. Inspired by these biological machines, molecules and molecular complexes that can be assembled to form rotors and motors have been designed and fabricated at the nanoscale to simulate biological functions. A motor is commonly defined as an energy converter system, i.e., a system capable of converting one type of energy—e.g., electromagnetic, chemical, electrical—into kinetic energy. Specifically, a rotary motor can convert this energy into unidirectional rotary movement in a controlled manner. Rotary molecular motors are commonly composed of three components: the rotor, the axle and the stator [15,59,66,67,68,69]. Research on rotary molecules has been developed almost over the last two decades, with increasing focus on systems anchored to solid surfaces. In 2005, Feringa and co-workers [66] envisioned that unidirectional rotors could be assembled as anchored molecular motors. The motors, developed in solution and anchored to gold nanoparticles, were composed of a helical alkene as a rotor, a carbon-carbon double bond as the axle and two thiol-functionalized legs that anchor to a gold stator. The authors introduce here a system in which conformational molecular changes on the rotor are driven by light and consequently induce unidirectional (360${}^{{}^{\circ}}$) rotational movement. This publication led to further studies within the field in which movement was brought upon by different stimuli and in other media (e.g., solution, vacuum and at interfaces), i.e., fabrication of rotors at the nanoscale could be tuned using (i) radiation, (ii) electric fields induced by the STM tips and even (iii) oxidation-reduction reactions [70,71].

**Figure 8:**
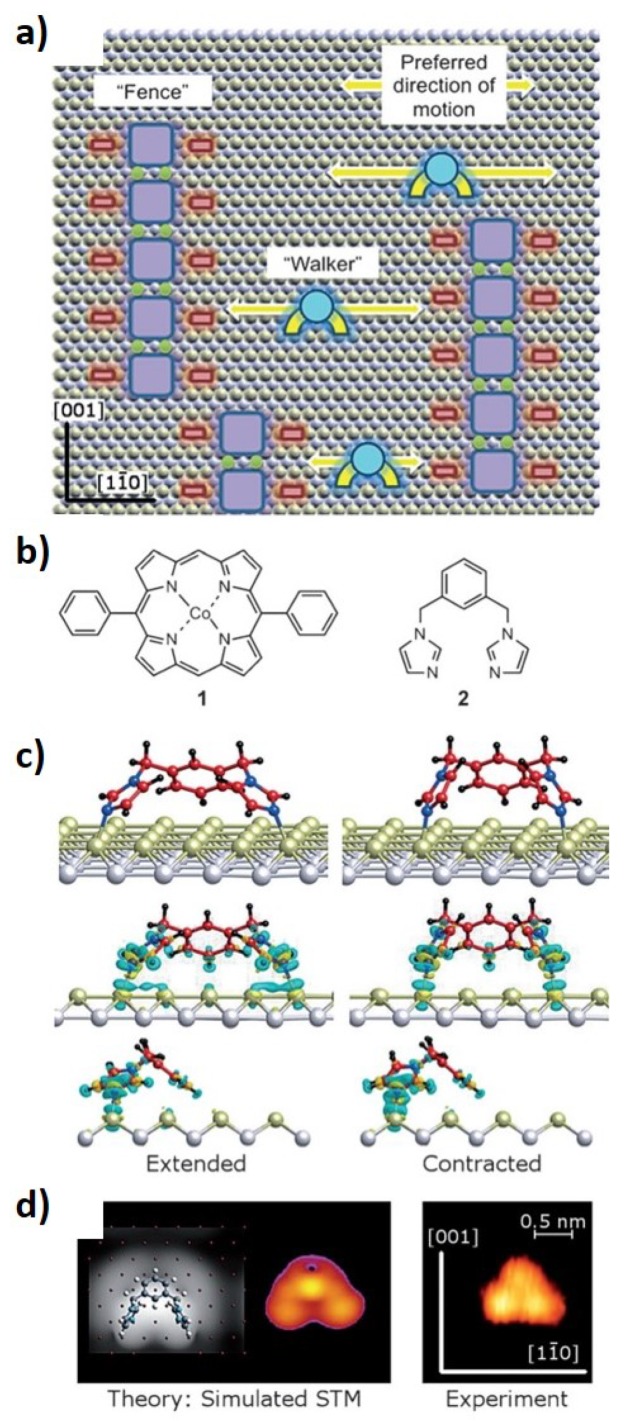
(**a**) Schematic representation of a walking molecule—a walker—which moves between two immobilized fences. The walker is a divalent bis(imidazolyl) molecule and the fence is a cobalt porphyrin represented in (**b**). The walker moves by attachment and detachment cycles between two fences, as represented by DFT simulations in (**c**), and the respective experimental STM image (**d**). (Reprinted with permission from reference [73]. Copyright 2015 WILEY-VCH Verlag GmbH & Co. KGaA, Weinheim.)

In the field of molecular motors, the focus has been on studies at room temperature and some important contributions have been published with dynamic molecular systems under these conditions [71,72,73,74,75]. A recent work [73] reports on a walking molecule moving freely at room temperature between two stationary points. The authors analyzed the movements of a divalent bis(imidazolyl) molecule, that they called a “walker”, which moves with an inchworm mechanism by attachment and detachment cycles between two immobilized cobalt porphyrins. Figure 8 shows a schematic representation of this system with a walker moving on an anisotropic Cu(110) surface. Density functional theory (DFT) calculations (Figure 8c,d) demonstrated that the imidazole adopts a “horseshoe conformation” characterized by the phenyl ring being pulled upwards and the two imidazolyl groups, which are in contact with the surface, work as the feet of the molecule. These imidazolyl groups are responsible for the movement through attachments and detachments. The walker moves freely through two opposite porphyrins. The system is thermally activated at room temperature and the authors suggest that it is a good candidate for molecular machines controlled by light or electrical pulses.

The development of molecular motors needs to overcome various barriers. Apart from their stability at room temperature, the focus is to develop fast machines able to “walk” over longer distances. To explore how fast and how far a nanomotor can travel, the first world-wide nanocar competing event took place using the STM to control and monitor the results [76,77,78]. The competition came to pass in the CEMES-CNRS center, in Toulouse, with six competing nanocars, over the span of two and a half days. The STM tip was employed as an external stimulus to drive the cars by applying an electrical voltage. All races were performed under ultrahigh vacuum, at a temperature of 4 K and over gold surfaces, apart from one which was performed over silver. It was stipulated that each nanocar should perform at least two laps in a 100 nm circuit. The results and details are described by Rapenne and Joachim in reference [78]. The winners were Rice–Graz and Basel whose car took 29 h to run across 1 μm. This can be the most impressive experiment with molecular machines that involved the collaboration of several international research teams. Via these experiments we can improve our understanding on the requirements to design robust molecular machines that can operate over long periods.

## 4. Molecular Wires

The first definition of a molecular wire (MW) was given by the winner of the Nobel Prize in Chemistry Jean-Marie Lehn in 1988, that defined this structure as a conducting chain of a molecule or a supramolecule which conducts electrons between two electrodes [79]. The MW is an important piece of a molecular electronic circuit considering that it can connect the single molecule elements. The charge transport of MWs has been studied by STM, using the tip as the top electrode and the substrate as the bottom electrode. The tip provides an individual electrical measurement of the wire. The charge transport depends on the interactions between the molecular or supramolecular system and the electrodes. All existing methods to measure individual properties have limitations and it is still a challenge to establish a method that gives real information about charge transport of MW. The most used techniques are current-sensing atomic force microscopy (CSAFM) and STM break junctions (STM BJ). Both techniques require physical contact between tip and MW. However, most of the time, the size of the tip that is used in CSAFM is greater than the molecule size and it is impossible to measure an individual MW without the influence of neighboring molecular systems. In the STM BJ the existing chemical contact between tip and molecule provides a great advantage [80]. A recent example of MWs that work as molecular switches in aqueous environments shows how it is possible to tune a single-molecule-based pH sensor electrically by STM break junctions [81]. The authors measured the electrical properties of 4,4${}^{\prime }$-vinylenedipyridine (44VDP) in a solution with 0.05 M of using Ni contacts. They monitored redox reactions at single molecule level controlling the pH of environment and the electrochemical potential. They observed a dependence of the electrical properties on the pH of the solution. The conductance switching of Ni|44VDP|Ni molecular junctions is due to redox reactions which occur at the end capping groups and that change the conductance. These molecular systems can be used as a pH-sensitive switch and the pH at which the switching event occurs can be tuned using a gate voltage. This is an example of how STM can control molecular conductance through the management of pH and bias voltage.

The conductance measurements at single molecule level are experimentally challenging and each system needs specific experimental protocols. The STM BJ experiments seem to be perfect taking into account that the electrodes make a contact with the molecular system. However, it is difficult to know how this affects the structure and the vibrational properties of the structure that is being analyzed.

Scanning Tunneling Spectroscopy (STS) is another technique to measure electrical properties of individual wires. Its main limitation is the absence of contact between the tip and the MW that compromises the accuracy of the measurements. However, STS can be supported by STM images that can be acquired simultaneously with current measurements. This allows the precise localization of MWs ensuring that the tip is analyzing the desired structure. The most reported works about MWs are related to supramolecular structures previously synthesized in laboratory or locally synthesized in high vacuum conditions [82].

**Figure 9:**
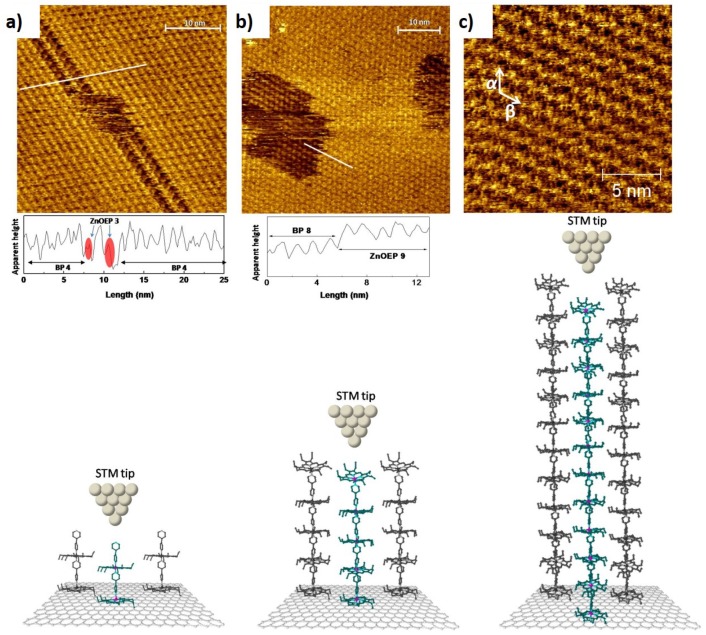
STM images of bipyridine monolayers assembled in intercalated structures with ZnOEP: (**a**) represents the fourth self-assembled monolayer (BP) developed over the third layer (darker regions—ZnOEP); (**b**) the ninth layer (ZnOEP) developed over the eighth layer (darker regions—BP), with corresponding line profiles evidencing the height differences in the monolayer; and (**c**) the twenty-third layer (ZnOEP). The schematics immediately bellow the STM images are models of the BP-ZnOEP alternating self-assembled layer-by-layer. (Reprinted with permission from reference [8]. Copyright 2014 American Chemical Society).

A recent work [8] shows how it is possible to use a combination of STM at the solid/liquid interface and STS to build a MW and to measure its individual electrical properties at room temperature. Vertical MWs with 14 nm of length were built using a stepwise method from 23 molecules added one by one. Zinc (II) octaethylporphyrins (ZnOEP) assembled on HOPG were bonded by 4,4${}^{\prime }$-bipyridine (BP) via coordination of the nitrogen atom to the zinc atom. ZnOEP and BP are two building blocks which were added sequentially until achieving the desired structure. These experiments were made at the HOPG/tetradecane interface, imaging each step with STM to control the formation of each molecular bond. Figure 9 shows a schematic representation of MWs composed of 4, 9, and 23 building blocks and the respective STM images. When observing the image of Figure 9a it is possible to see, at high resolution, the 4th monolayer with a defect that allows the visualization of the monolayer immediately below. Each brighter spot corresponds to the top of a MW composed of 4 molecules, covalently linked: ZnOEP/BP/ZnOEP/BP. The respective line profiles highlight the presence of the last molecule (BP) and the ZnOEP before that. In this work, the authors reported on the STM imaging of every step maintaining the resolution until the last monolayer, shown in Figure 9c. The electric characteristics of individual wires were also quantified by the authors using STS during the scanning of each monolayer.

The imaging allows the positioning the tip at the top of a single wire. At this moment, the individual current is measured by the application of a bias voltage between the tip and the substrate. The characterization of the conduction mechanisms is identified analyzing the evolution of the MW electrical resistance as a function of the molecular length, associating a tunneling mechanism for shorter wires with 4–5 nm and a hopping mechanism for longer wires. This work can be taken as a model to build similar supramolecular structures synthesized in situ. Each step can be controlled by imaging with molecular resolution while measuring, at the same time, the individual electrical transport.

The described stepwise method opens the way for the design of a variety of MWs by changing the building blocks to optimize the conductive properties.

## 5. Perspectives

One of the questions that we may ask when looking at the developments of unimolecular devices is “when will they be integrated in electronic circuits?” The answer can be foreseen by the fast evolution in this research field covering a wide range of applications and prototypes which are already working [83,84,85,86,87,88]. Organic sensors, organic solar cells, organic transistors, organic light-emitting diodes are no longer future projects and they are already existing in our days. The breakthrough for the development of organic devices is to develop smart devices able to replace silicon semiconductor components. The trend is to learn with biological systems [89,90,91,92,93,94,95]. To develop functional devices, it is important to understand how biological machines work and mimic their mechanisms. At first instance, this could be an easy task considering that we have 23 building blocks in nature corresponding to the amino acids. However, most of the mechanisms of biological systems remain unknown. The focus has been centered in studying individual biological molecules [93,94]. STM has been a fundamental tool to study biosystems in aqueous environment [96]. The characterization of electrical properties of biomolecules can help us to design bioelectronic devices. Charge transport characteristics of redox proteins have been studied in nanodevices. For example, Cu-azurin was analyzed in microscale solid-state devices [97,98,99]. Namely, single-protein junctions of Cu-azurin were performed with STM and showed that it is possible to manipulate conformations preserving the protein structure [94].

Besides the interest in biological systems, there are other challenges to overcome:

- to create unimolecular systems stable at room temperatures;
- to develop encapsulation methods while maintaining the integrity of the organic device.

A recent work, shows how it is possible to build flexible MWs stable at room temperature [100]. MWs with approximately 4 nm of length composed of disulfanyl carbon-bridged oligo-(phenylenevinylene) (COPV${}_{6}$(SH)${}_{2}$) maintained their electronic characteristics at room temperature for 3 months and a high tolerance for low and high temperatures. The authors explained that this behaviour is due to the structural characteristics of COPV${}_{6}$ which is a rigid and planar molecular system composed of a conjugated wire which is protected by an insulating sheath. Consequently, the electron flow is stable and does not suffer influences from other neighbouring molecular systems. This can be the departure point to optimize these systems and implement them in electronic circuits.

The stability of an organic device also depends on the encapsulation and this research is still at the beginning. All devices need to be encapsulated to be protected from corrosion and to avoid degradation in the presence of air. L. Moro et al. published a recent article in which he gives an overview of the requirements to develop encapsulation methods for organic devices [101]. This is a research field which needs a greater dedication and work to accomplish the fast development of devices.

At the moment, the main concern of scientists is to integrate the single molecule device in a final electronic circuit. To achieve this goal, it is necessary to overcome important constraints: long-term stability, biocompatibility, and environment effects.

## Figures and Tables

**Table 1:** Table summarizing the three groups of building blocks used to form nanoporous self-assembled monolayers.

Nanoporous Self-Assembled Networks			
Bonding between Building Blocks	Building Blocks	Range of Pore	References
Hydrogen bonds	TMA	1–2.8 nm	[41,42]
van der Waals interactions	DBA	1.6–4.7 nm	[43,44,46,50]
Metal-Ligand coordination bonds	Dicycanooligophenylene	4.2–6.7 nm	[48,49]
